# Rosmarinic acid suppresses Alzheimer’s disease development by reducing amyloid β aggregation by increasing monoamine secretion

**DOI:** 10.1038/s41598-019-45168-1

**Published:** 2019-06-18

**Authors:** Tomoki Hase, Syun Shishido, So Yamamoto, Rei Yamashita, Haruka Nukima, Shu Taira, Tsudoi Toyoda, Keiko Abe, Tsuyoshi Hamaguchi, Kenjiro Ono, Moeko Noguchi-Shinohara, Masahito Yamada, Shoko Kobayashi

**Affiliations:** 1 Research Center for Food Safety, Graduate School of Agricultural and Life Sciences, The University of Tokyo, Yayoi, Bunkyo-ku, Tokyo, 113-8657 Japan; 2 Faculty of Food and Agricultural Sciences, Fukushima University, Kanayagawa, Fukushima, 960-1248 Japan; 3 Department of Applied Biological Chemistry, Graduate School of Agricultural and Life Sciences, The University of Tokyo, Yayoi, Bunkyo-ku, Tokyo, 113-8657 Japan; 4 Group of Food Functionality Assessment, Kanagawa Institute of Industrial Science and Technology, Life Science Environment Research Center, Tonomachi, Kawasaki, Kanagawa, 210-0821 Japan; 5 Department of Neurology and Neurobiology of Aging, Kanazawa University Graduate School of Medical Sciences, Takara-machi, Kanazawa, 920-8640 Japan; 6 Division of Neurology, Department of Medicine, Showa University School of Medicine, Hatano-dai, Shinagawa-ku, Tokyo, 142-8666 Japan

**Keywords:** Cognitive ageing, Blood-brain barrier, Alzheimer's disease

## Abstract

A new mechanism is revealed by which a polyphenol, rosmarinic acid (RA), suppresses amyloid β (Aβ) accumulation in mice. Here we examined the brains of mice (Alzheimer’s disease model) using DNA microarray analysis and revealed that the dopamine (DA)-signaling pathway was enhanced in the group fed RA versus controls. In the cerebral cortex, the levels of monoamines, such as norepinephrine, 3,4-dihydroxyphenylacetic acid, DA, and levodopa, increased after RA feeding. The expression of DA-degrading enzymes, such as monoamine oxidase B (Maob), was significantly downregulated in the substantia nigra and ventral tegmental area, both DA synthesis regions. Following *in vitro* studies showing that monoamines inhibited Aβ aggregation, this *in vivo* study, in which RA intake increased concentration of monoamine by reducing *Maob* gene expression, builds on that knowledge by demonstrating that monoamines suppress Aβ aggregation. In conclusion, RA-initiated monoamine increase in the brain may beneficially act against AD.

## Introduction

Alzheimer’s disease (AD) is a neurodegenerative disease causing cognitive dysfunction such as memory impairment and disorientation. The main pathological hallmark of AD is presence of neuritic plaques arising from accumulation of extracellular amyloid β (Aβ) protein. Two kinds of AD therapeutic agents are available: cholinesterase inhibitors (e.g., donepezil, galantamine, and rivastigmine) and NMDA receptor antagonists (e.g., memantine)^1,2^. These drugs are able to ameliorate symptoms but cannot entirely suppress them. For this reason, prevention, early detection, and early treatment of AD are crucial.

From a dietetic point of view, our research is focusing on a food-borne polyphenol as one strategy for AD prevention. Many researchers have reported that polyphenols may be useful as therapeutic molecules against AD^3–7^. In a previous *in vitro* study, we screened five polyphenols and among those found that rosmarinic acid (RA), ferulic acid (FA) and curcumin (Cur) significantly inhibited Aβ aggregation^8^. We further investigated these five polyphenols in an *in vivo* study using Tg2576 mice as an AD model and found that RA showed the highest Aβ aggregation inhibitory activity in the brain^9^. RA is a polyphenol notably found in the *Lamiaceae* including rosemary and lemon balm, possessing antioxidant and anti-inflammatory effects^10–12^. The mechanism of inhibition of Aβ aggregation *in vitro* has been analyzed by our group^13^. Nuclear magnetic resonance imaging and western blotting demonstrated that RA binds directly to Aβ and inhibits aggregation by preventing the β-sheet structure formation during the aggregation process. Polyphenols are known to have a variety of physiological activities, however, and in general the intestinal permeability of orally ingested polyphenols is low. For example, it has been reported that the permeabilities of Cur and RA are approximately 0.1% and 1%, respectively^14,15^. In addition, due to the blood–brain barrier (BBB) created by brain capillary endothelial cells, the brain is strictly regulated so that exogenous materials such as polyphenols in the blood do not pass into the brain. All these factors would appear to indicate that RA taken orally would be absorbed and then excreted in a relatively short time, reaching the brain only with difficulty. In the *in vivo* tests used in this study, Tg2576 mice were fed with a diet including 0.5% RA, but it was considered unlikely that RA concentration in the brain would reach sufficient concentration, therefore it was inferred that its suppression effect on Aβ aggregation *in vivo* arose through other mechanisms. We focused on the probable new mechanism of suppression of Aβ accumulation following RA feeding.

## Results

### Low permeability of RA into the brain

**Figure 1:**
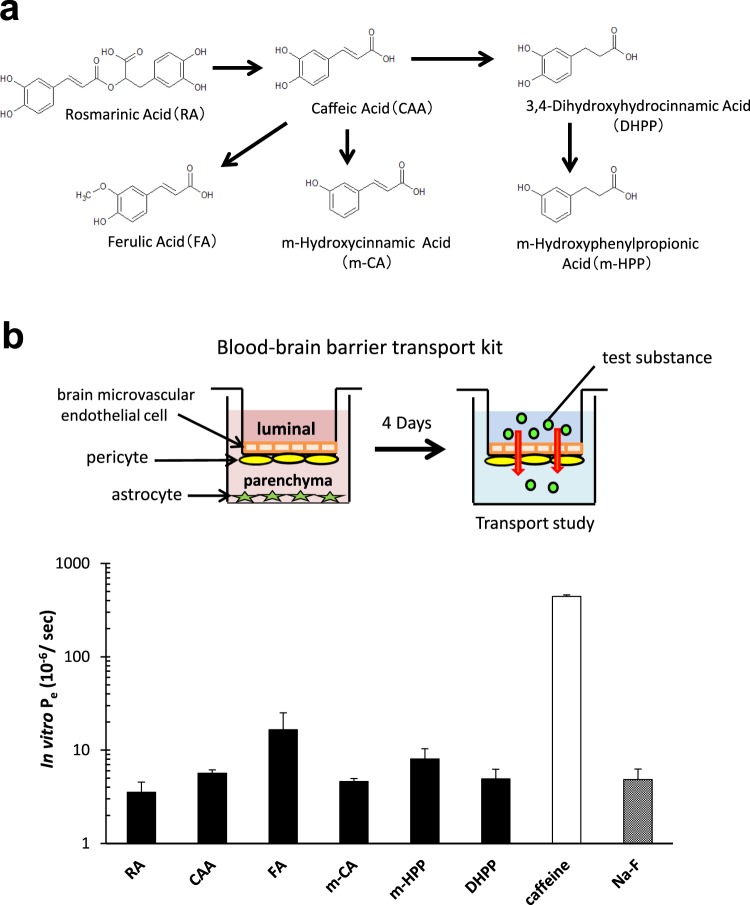
BBB permeability of RA and its metabolites. (**a**) Proposed metabolic pathway of RA administrated orally. (**b**) Measurement of transendothelial permeability (Pe) in brain capillary endothelial cell monolayers using an *in vitro* BBB model. Caffeine and sodium fluorescein (Na-F) were used as positive and negative controls, respectively. Results are expressed as mean ± S.E. (n = 3). These data indicate that RA permeability is very low.

In a previous *in vitro* study, we had confirmed that the intestinal permeability of RA is <1% of its intake volume^15^. In this study, to discover whether orally administrated RA transfers into the brain, we used high-performance liquid chromatography coupled with electrochemical detection (HPLC-ECD) to measure the concentration of RA and its metabolites (arising from the gut microbiota) in the brain and plasma of wild type mice fed a diet containing RA for 7 weeks. RA was detected in the plasma but not in the brain, regardless of β-glucuronidase treatment (Table 1), while RA metabolites were not detected at all (data not shown). The detection limit for RA was <0.01 ppm using HPLC-ECD analysis. We have proposed metabolic pathways for RA taken orally (Fig. 1a)^15^. Further, to examine whether RA and its metabolites are able to pass through the BBB into the brain, we measured their permeability using an *in vitro* BBB model (Fig. 1b). Results showed that their rates of permeability were all relatively similar to that of a negative control, sodium fluorescein (Na-F) (Fig. 1b), with RA showing the lowest value among the six test compounds. These data suggest that the intestinal permeability of RA is very low, and it migrates to the brain with difficulty due to the presence of the BBB. Therefore, it is likely that the mechanism by which RA suppresses the accumulation of Aβ plaques is not only via direct binding but also through some other mechanism.

**Table 1:** Concentration of RA in the brain and plasma of C57Bl/6 J mice fed RA for 7 weeks.

	β-glucuronidase	plasma (μmol/L)	brain (μmol/L)
RA	+	0.75 ± 0.15	N.D.
	−	0.59 ± 0.22	N.D.
C	+	N.D.	N.D.
	−	N.D.	N.D.

+: β-glucuronidase reacted, −: β-glucuronidase not reacted.

Wild type mice fed with 0.5% RA for 7 weeks were sacrificed. Brain and plasma samples were taken and treated appropriately, and the concentration of RA was measured by HPLC-ECD (in which the limit of detection of RA is <0.01 ppm). RA was detected in plasma but not in brain. N.D.: not detected. Values are expressed as mean ± S.E. (n = 5).

### Upregulation of dopamine secretion and the dopaminergic synapse pathway as suggested by DNA microarray analysis

To clarify the mechanism of suppression of Aβ plaque accumulation by RA, we performed DNA microarray analysis in the brain of Tg2576 mice fed either a control diet or a diet containing 0.5% RA for 10 months. The 367 probe sets were upregulated and the 636 probe sets were downregulated in the RA group compared with the control group in terms of differentially expressed genes (DEGs), which satisfied the criterion of a false discovery rate (FDR)^16^ <0.05.

First, to obtain an overview of and to classify DEG function, we used the Database for Annotation Visualization and Integrated Discovery (DAVID). We annotated these DEGs with gene ontology (GO) categories (FDR <0.05) and arranged the categories hierarchically using QuickGO (Table 2). DEGs were annotated with GO terms related to nervous system development (e.g., nervous system development and brain development), neurotransmission (e.g., chemical synaptic transmission and neurotransmitter secretion), and memory (e.g., long-term memory). These data suggested that nervous system development, neurotransmission, and memory function changed in the RA group.

**Table 2:** GO analysis of DEGs in brains of Tg2576 mice fed the RA diet for 10 months.

GO ID	GO term	annotated gene number
GO:0007399 GO:0007420	nervous system development brain development	52 35
GO:0007268 GO:0007269	chemical synaptic transmission neurotransmitter secretion	28 10
GO:0007616	long-term memory	11
GO:0006351 GO:0006355 GO:0045893 GO:0045944	transcription, DNA-templated regulation of transcription, DNA-templated positive regulation of transcription, DNA-templated positive regulation of transcription from RNA polymerase II promoter	172 206 80 113
GO:0000122	negative regulation of transcription from RNA polymerase II promoter	79
GO:0008285 GO:0050680	negative regulation of cell proliferation negative regulation of epithelial cell proliferation	49 17
GO:0001568	blood vessel development	16
GO:0007507	heart development	17
GO:0045778	positive regulation of ossification	37
GO:0048839	inner ear development	21
GO:0090090	negative regulation of canonical Wnt signaling pathway	9

DNA microarray analysis was conducted on the brains of Tg2576 mice fed the RA diet for 10 months. DEGs were identified with GO terms such as nervous system development, brain development, chemical synaptic transmission, neurotransmitter secretion, and long-term memory (FDR < 0.05).

**Figure 2:**
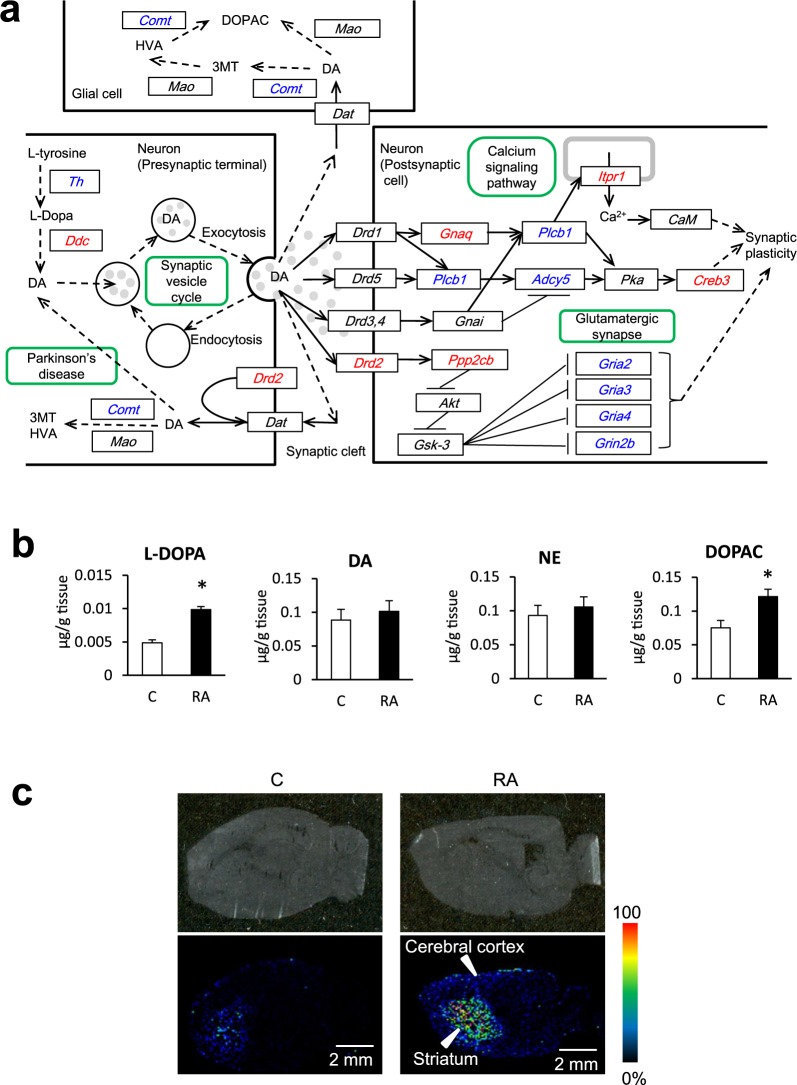
Enhanced dopaminergic synapse pathway as deduced by KEGG analysis in the brain of Tg2576 mice fed an RA-supplemented diet for 10 months and monoamine concentrations in the brain 7 weeks and 11days after RA intake. (**a**) The pathway of “dopaminergic synapse” in KEGG pathway. Red and blue gene symbols framed black represent genes that were significantly up- and downregulated, respectively. Green frame: map. Arrow: expression, activation, and molecular interaction or relation. Dashed arrow: indirect effect or state change. Tg2576 mice express a spliced form of 695 amino acid residues of the human amyloid precursor protein modified by the Swedish Familial AD double mutation K670N-M671L. The mice were divided into a control group (n = 10) fed a normal diet, and the RA group (n = 10) fed an RA diet for 10 months. DA secretion to the synaptic cleft and the Drd2 dopaminergic synapse pathway were upregulated in the RA group. (**b**) Concentration of L-DOPA, DA, NE, and DOPAC in the brain of mice fed with 0.5% RA for 7 weeks. Results are expressed as mean ± S.E. (n = 5) *t*-test: *P < 0.05 vs. control group. L-DOPA and DOPAC were significantly increased. (**c**) MALDI-TOFMS imaging of DPP-derivatized DA in the brains of mice fed the 0.5% RA diet for 10 days. Relative abundance and distribution of DA in a sagittal tissue section by IMS. The obtained image data are presented using a rainbow scale. The signals, which represent DA, were observed more strongly in the cerebral cortex and striatum region of mice in the RA diet group than in the control group.

Second, to identify significantly enriched pathways, we conducted Kyoto Encyclopedia of Genes and Genomes (KEGG) pathway analysis in DAVID. The top 10 KEGG pathways are shown in Table 3. We focused on the “dopaminergic synapse” pathway because the levels of dopamine (DA)-related monoamines, such as levodopa (L-DOPA), DA, 3,4-dihydroxyphenylacetic acid (DOPAC), norepinephrine (NE), and 5-hydroxytryptamine (5-HT), have been reported to decrease in the brains of AD patients^17–20^. The KEGG pathway for “dopaminergic synapse” is shown in Fig. 2a. The genes encoding the DA synthesis enzyme DOPA decarboxylase (*Ddc*) and dopamine receptor D2 (*Drd2*) were upregulated, and the gene for the DA degradation enzyme catechol-*O*-methyltransferase (*Comt*) was downregulated in presynaptic terminal neurons (Fig. 2a). These data suggested that DA biosynthesis and secretion from presynaptic terminal neurons was increased in the RA group. In postsynaptic cell neurons, *Drd2* and the gene for protein phosphatase 2 (formerly 2 A), catalytic subunit, β isoform (*Ppp2cb*) were upregulated and, as a result, the downstream glutamatergic synapse-related genes, such as glutamate receptor, ionotropic, AMPA2, 3, and 4 (alpha 2, 3, and 4) (*Gria2, Gria3*, and *Gria4*), and glutamate receptor, ionotropic, NMDA2B (epsilon 2) (*Grin2b*) were downregulated. These results suggested that *N*-methyl-D-aspartate receptor (NMDAR) activity was decreased in the RA group. Excessive NMDAR activity causes excitotoxicity and promotes cell death, underlying a potential mechanism of neurodegeneration occurring in AD^21^. These changes in NMDAR-related genes indicate changes in synaptic plasticity.

**Table 3:** Top 10 KEGG pathways of DEGs in the brain of Tg2576 mice fed a normal or RA-supplemented diet for 10 months.

Pathway ID	KEGG pathway	annotated gene number
mmu05030	Cocaine addiction	13
mmu04724	Glutamatergic synapse	20
mmu04015	Rap1 signaling pathway	29
mmu04918	Thyroid hormone synthesis	14
mmu05202	Transcriptional misregulation in cancer	24
mmu05200	Pathways in cancer	45
mmu04728	Dopaminergic synapse	20
mmu04540	Gap junction	15
mmu04024	cAMP signaling pathway	26
mmu04713	Circadian entrainment	16

Ten KEGG pathways were selected in ascending order of modified Fisher’s exact test P value.

We focused on the “dopaminergic synapse” pathway because levels of DA-related monoamines (such as L-DOPA, DA, DOPAC, NE, and 5-HT) have been reported to decrease in the brains of AD patients.

Since the DNA microarray results suggested that DA secretion and the dopaminergic synapse pathway were upregulated in the RA group, we subsequently focused on the DA-related pathway.

### RA administration increases monoamines in the brain

We first measured monoamine concentration in the whole brain of mice fed the RA diet for 7 weeks. L-DOPA and DOPAC concentrations were significantly increased (Fig. 2b) while those of DA and NE changed but not significantly. Thereafter, with the aim of elucidating the brain region where monoamine levels increased, imaging mass spectrometry (IMS) was used to visualize the localization of monoamine. Since only DA could be derivatized, we acquired the imaging data of 2,4-diphenyl-pyranylium tetrafluoroborate (DPP)-derivatized DA. Signals from the brains of mice in the RA diet group (representing DA) were observed in the cerebral cortex and striatum regions more strongly than in control group (Fig. 2c).

**Figure 3:**
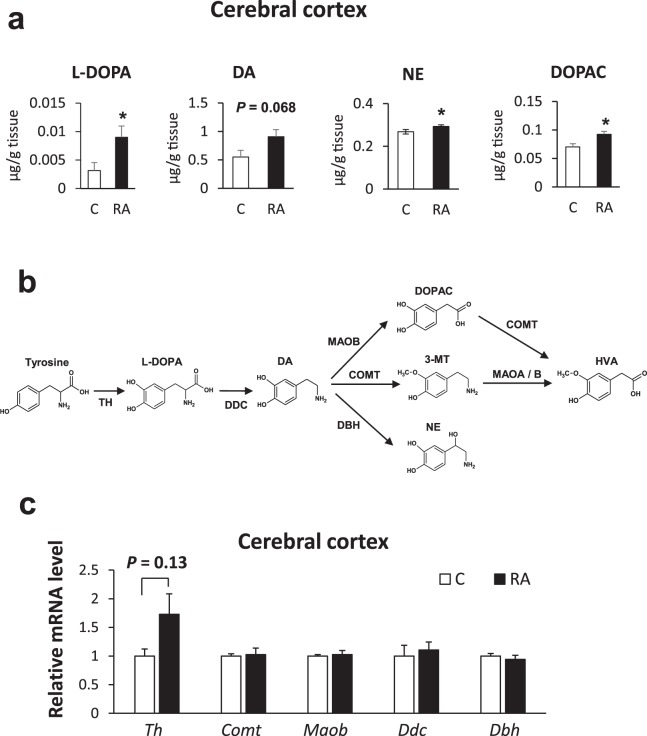
Monoamine concentration and mRNA expression levels of DA-metabolizing enzymes in the cerebral cortex of mice after 11 days RA intake. (**a**) Levels of L-DOPA, DA, NE, and DOPAC in the cerebral cortex of mice fed with 0.5% RA for 11 days. Results are represented as mean ± S.E. (n = 5) t-test: *P < 0.05 vs. control group. L-DOPA, NE, and DOPAC levels were raised significantly in the cerebral cortex. (**b**) DA-metabolizing pathway. L-DOPA is produced from tyrosine by TH, and DA is synthesized from L-DOPA by DDC. DA is metabolized to NE, 3-MT, or DOPAC by *Dbh*, *Comt*, and *Maob*, respectively. DOPAC and 3-MT are metabolized to HVA by *Comt* and *Maoa/b*, respectively. TH is the rate-limiting enzyme in this pathway. (**c**) Effect of RA intake for 11 days on the DA-metabolizing enzyme mRNA levels in the cerebral cortex. We conducted qRT-PCR analysis of DA-metabolizing enzyme mRNA levels in the cerebral cortex of mice fed RA for 11 days. No significant changes in expression were confirmed. Data are normalized to GAPDH. Results are expressed as mean of fold change compared with control condition. (S.E as error bars, C: n = 5, RA: n = 6).

In order to examine the effect of a single dose of RA, we conducted intragastric administration of RA at 0.1 m mol/kgBW to wild type mice and measured the monoamine concentration in three brain regions (cerebral cortex, striatum, and hippocampus) after 0, 60, and 240 min. Monoamine concentrations showed no significant differences between the three brain regions investigated (data not shown). From these results we concluded that a single administration of RA was not useful for revealing monoamine effects. It has been reported that the concentrations of NE, DA, and 5-HT are significantly enhanced in the cerebral cortex of ICR mice treated for one week with rosemary extract containing RA^22^. Therefore, we extended the RA administration period to 11 days. Measurements of monoamine concentration in each brain region revealed that L-DOPA, NE, and DOPAC levels increased significantly (*P* < 0.05) while the DA level was somewhat increased (*P* = 0.068) in the cerebral cortex when compared with controls (Fig. 3a). In the striatum and hippocampus, the monoamine concentration was not significantly changed in the RA diet group (data not shown).

### Upregulated tyrosine hydroxylase gene and downregulated dopaminergic enzyme genes

In the DA metabolic pathway, tyrosine hydroxylase (Th), which produces L-DOPA from tyrosine, is considered to be the rate-limiting enzyme. DA is produced from L-DOPA by DDC which is then changed to NE, 3-methoxy-4-hydroxyphenethylamine (3-MT) or DOPAC by dopamine β-hydroxylase (Dbh), Comt, and monoamine oxidase B (Maob), respectively (Fig. 3b). To clarify the mechanisms of monoamine level increases caused by RA, we first measured relative mRNA levels of DA-metabolizing enzymes in the cerebral cortex of RA-fed mice using quantitative real-time polymerase chain reaction (qRT-PCR). The results showed no significant difference in expression levels (Fig. 3c), though *Th* was somewhat increased (*P* = 0.13). These data indicate that RA administrated for 11 days slightly influenced gene expression of *Th* in the cerebral cortex.

**Figure 4:**
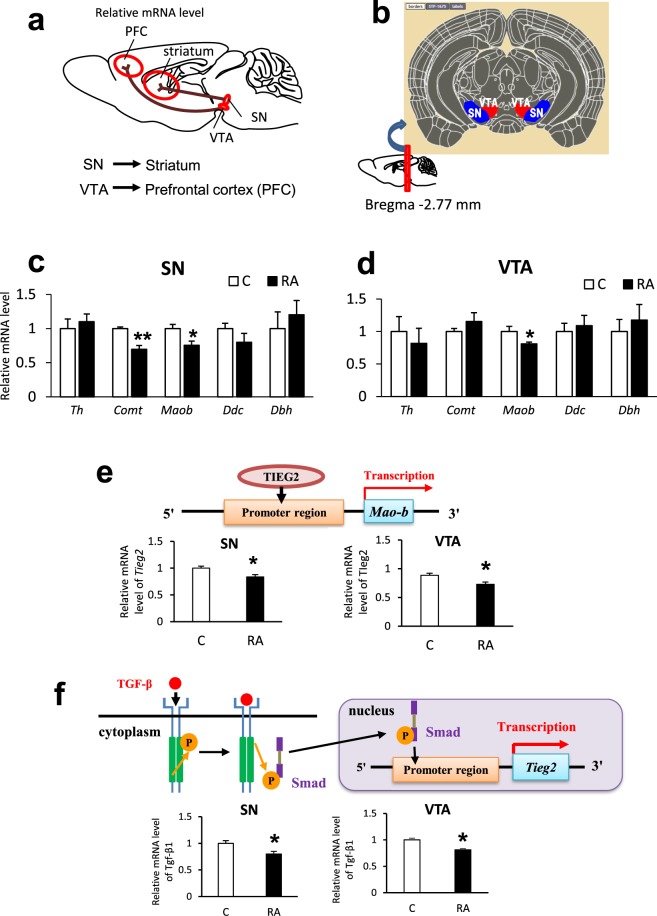
DA projection pathway and mRNA expression levels of DA-metabolizing enzymes in SN and VTA and their transcription factor of *Tieg2* and *Tgf-β1*. (**a**) DA is projected from substantia nigra (SN) to striatum, and from ventral tegmental area (VTA) to prefrontal cortex (PFC) or nucleus accumbens (NAcc). (**b**) Section of the mouse brain subjected to qRT-PCR. (**c**,**d**) Effect of RA intake for 11days on DA-metabolizing enzyme mRNA levels. We conducted qRT-PCR analysis of DA-metabolizing enzyme mRNA levels in (**c**) SN and (**d**) VTA. *Maob* was downregulated in both SN and VTA, and *Comt* was downregulated in SN. (**e**) *Tieg2* mRNA levels were measured in SN and VTA of mice fed the RA diet for 11 days (C: n = 5, RA: n = 6). *Tieg2* was significantly downregulated in both SN and VTA. (**f**) *Tgf-β1* mRNA levels in SN and VTA of mice fed the RA diet for 11 days (n = 5). *Tgf-β1* was significantly downregulated in both SN and VTA. Data are normalized to β-actin. Results are expressed as mean of fold change compared with control condition. (S.E as error bars, C: n = 5, RA: n = 6) t-test: **P < 0.01, *P < 0.05 vs. control group.

Brain DAergic neuron projection mainly occurs along two pathways (Fig. 4a). One is from substantia nigra (SN) to striatum, the other is from ventral tegmental area (VTA) to cerebral cortex^23^. We focused on the DA synthesis region and measured relative mRNA levels of DA-metabolizing enzyme in the SN and VTA of RA-fed mice (Fig. 4a,b). The expression of *Comt* was significantly downregulated in the SN region, and so was that of *Maob* in both SN and VTA regions (Fig. 4c,d). These data suggest that the increasing DA-related monoamine levels in the cerebral cortex is caused by significantly decreased expression of DA-degrading enzymes in the DA synthesis regions, and DA-related monoamines are quickly transported to the striatum and cortex after production in SN and VTA.

### Decreased expression of *Maob-*related transcription factor

The *Transforming Growth Factor-β (Tgf-β)-Inducible Early Growth Response Protein 2* (*Tieg2*) gene is in the promoter region of *Maob* (Fig. 4e), and it has been reported that *Tieg2* activates *Maob* gene expression^24,25^. We measured relative *Tieg2* mRNA levels in the SN and VTA of RA-fed mice and found that *Tieg2* gene expression was significantly decreased in both regions compared with controls (Fig. 4e). This result suggests that *Tieg2* is one of the factors involved in regulation of *Maob* expression following RA administration.

Transforming growth factor β is one of the cytokines that regulates cell proliferation and apoptosis; it has been reported that *Tgf-β* signaling upregulates *Tieg2* expression^26^ (Fig. 4f). Measurement of relative mRNA levels by qRT-PCR revealed that *Tgf-β1* gene expression was significantly downregulated in the SN and VTA of mice fed the RA diet for 11 days (Fig. 4f). Several studies have reported that RA suppresses the expression of *Tgf-β1 in vivo* and *in vitro*^27,28^. We conclude that the down-regulation of *Tgf-β1* in the SN and VTA after RA intake for 11 days leads a decrease *Tieg2* expression, and subsequent down-regulation of *Maob*, resulting in an increase in the DA-related monoamine levels in the cerebral cortex and striatum.

### Suppression of Aβ aggregation by DA and other monoamines

**Figure 5:**
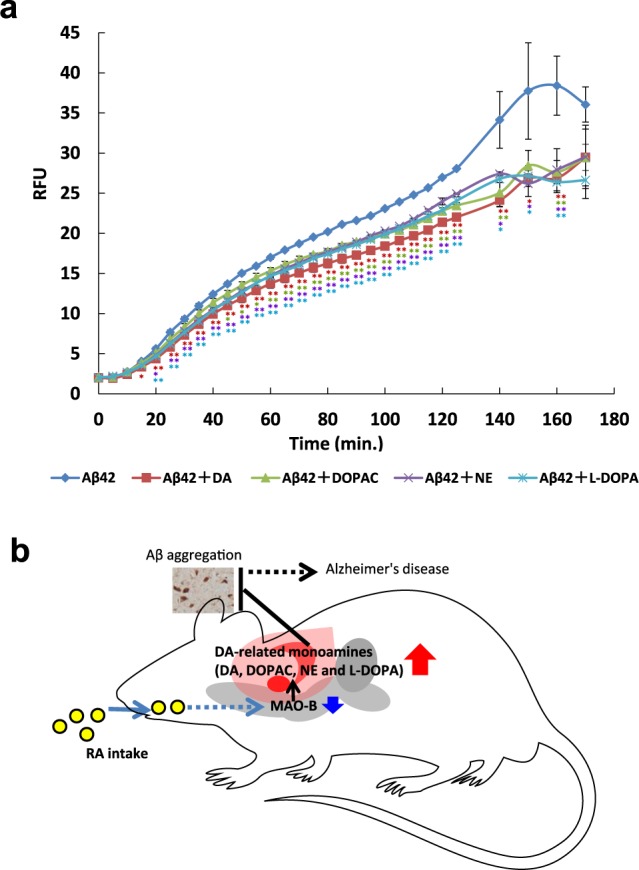
Inhibition of Aβ aggregation by monoamines and schematic representation of the mechanism by which accumulation of Aβ is suppressed in mice fed RA. (**a**)Effect of monoamines on Aβ aggregation was examined using SensoLyte Thioflavin T β-Amyloid Aggregation Kit. With Aβ alone, thioflavin T gradually bound and emitted fluorescence. DA, DOPAC, L-DOPA, and NE significantly (p < 0.05) inhibited Aβ aggregation at 15–160, 45–140 and 160, 20–160, and 20–160 min, respectively. Results are expressed as mean ± S.E. Statistical analyses were performed using Dunnett’s multiple comparisons test (*P < 0.05; **P < 0.01). (**b**) RA intake reduces *Maob* expression in SN and VTA, leading to increased levels of monoamines, such as L-DOPA, DA, DOPAC and NE, which inhibits Aβ aggregation.

It has been reported that DA and L-DOPA inhibit Aβ aggregation^29,30^. To examine this effect, we measured fluorescence intensity when monoamines were added to Aβ using a SensoLyte Thioflavin T β-Amyloid Aggregation Kit (Fig. 5a). As thioflavin T (ThT) binds with the β-sheet structure of Aβ fibrils, fluorescence intensity increases. All groups reached the plateau of the effect within 3 h. When DA, DOPAC, NE, and L-DOPA were added to Aβ, the relative fluorescence intensity was lower than that in their absence. These results indicate that DA, DOPAC, NE, and L-DOPA inhibit Aβ aggregation.

## Discussion

The present study shows that RA has low permeability into the brain *in vitro* and *in vivo*. We thus focused on the concentration of monoamines in the brain. Monoamines are neurotransmitters in which the amino group is connected to an aromatic ring by a two-carbon chain. They act as chemical transmitters in peripheral tissues, and as a hormone in the sympathetic nerves, but in the central nervous system, they mainly function as neurotransmitters. The relationship between DA and AD has been reported^19,20^. DA receptors are decreased in postmortem brain examinations of AD patients. It has also been reported that cognitive impairment accompanying AD is improved by enhancing DA neurotransmission in AD patients^18^. It has also been shown that due to aging, the release of DA from nerve endings is reduced, and the expression of DA receptors and transporters that recover DA released from the synaptic cleft to the presynaptic nerve decreases^31^. In addition, it has been shown that gene expression of Th (the rate-determining enzyme of DA production) is decreased in the midbrain of AD patients^32^. It has also been reported that DA decreases in the cerebrospinal fluid of AD patients, and it has been suggested that a decrease in DA may significantly influence AD^18^. In our study, RA intake for 11 days increased the DA level in the cerebral cortex and striatum. These data indicate that RA administration might prevent a decrease in the DA level in the brain of AD patients. It has been reported that the levels of NE, DA, and 5-HT are significantly elevated in the cerebral cortex of ICR mice treated for 1 week with rosemary extract containing RA^22^. In our study, we were able to observe the influence of RA in a pure form using a standardized RA product in the experimental diets.

There are several reports that monoamines in the brain increase following feeding with polyphenols. For example, quercetin, a type of flavonoid, suppressed a decrease in brain serotonin concentration via inhibition of monoamine oxidase A (Maoa; an enzyme that metabolizes serotonin)^33^. In mice fed theobromine for 20 days, significant increases in DA and NE concentrations in the striatum in association with elevated *Th* expression in the SN have been reported^34^. The expression of *Th* significantly increased in the cerebral cortex when rosemary extract containing 4% RA was orally administered for one week^22^. In this study, since there was limited change in *Th* expression following RA feeding, we considered that a new effect was involved, different from enhancement of DA synthesis. Therefore, we focused on *Maob* whose expression was significantly low in the SN and VTA.

Monoamine oxidase (MAO) is present mainly in the outer membrane of mitochondria, and in humans there are two types, MAOA and MAOB. The amino acid sequence of MAO is conserved across species. The main substrates of MAOA are NE or 5-HT, while that of MAOB is DA. It is known that the amount of MAOB is high in AD patients and patients with Parkinson’s disease (PD)^35^. In aged rats, the concentration of MAOB increases in the striatum and cerebral cortex^36^. This not only promotes degradation of DA but also produces reactive oxygen species such as hydrogen peroxide as a byproduct, which damages proteins, membrane lipids, and nucleic acids to cause dysfunction of nerve cells^37^. Meanwhile, it has been reported that *Maoa/Maob* double-knockout mice have lower body weight, increased anxiety-like behavior, heart rate abnormalities, etc., unlike wild type mice^38^. An excessive decline in *Mao* expression does not necessarily have positive influences, and it is considered important to maintain an appropriate expression balance. Selegiline, as a MAOB inhibitor, has been used for PD therapy. To our knowledge, this study is the first to report that *Maob* expression is influenced by dietary polyphenol.

TIEG2 is one of the transcription factor of MAOB, and its expression is regulated by interaction with the promoter region of MAOB (Fig. 5a)^23,24^. TIEG2 has two functions; it acts as a repressor for the CACCC region of the MAOB promoter, and also as an activator at the Sp1-binding site (it has been shown to interact directly with the CACCC and Sp1-binding sites in *in vitro* and *in vivo* studies)^39^. As a MAOB promoter, TIEG2 possesses a higher affinity for the Sp1-binding site than for the CACCC region and is therefore considered to increase mRNA expression^24^. TIEG2 is a type of TGF-β-inducible early growth response protein, and its transcription is activated by TGF-β^25^. The TGF-β receptor is phosphorylated after binding TGF-β (Fig. 5b), which is followed by phosphorylation of Smad and its translocation to the nucleus where it binds at the TIEG2 promoter region. TGF-β is a cytokine responsible for suppressing cell proliferation and promoting apoptosis^40^, and its expression is suppressed by RA^26,27^. In our study, the expression of *Tieg2* was significantly decreased in the SN and VTA. We suggest that as a result of RA administration, the expression of *Tgf-β* decreased and the expression of *Tieg2* was suppressed. Consequently, transcription activity of *Maob* decreased, and the degradation of DA was suppressed, resulting in the DA-related monoamine levels increasing in the cerebral cortex and striatum. The detailed mechanism by which RA intake increases monoamine levels in the brain is unknown. The possibility is that very low concentrations of RA or its metabolites suppress *Tgf-β*, *Tieg2*, and *Maob* expression. The brain transmits signals to the gut according to a finding previously reported that polyphenols and their metabolites transmit a signal to the brain via the vagus nerve^41^. This is also the possible reason why RA increases monoamine levels in the brain via the brain–gut axis system because a relatively high level of RA may exist in the gut after the feeding. A future study is needed to clarify the mechanisms.

We measured the inhibition of Aβ aggregation by monoamines (DA, DOPAC, NE, and L-DOPA) whose levels were elevated in the cerebral cortex of mice fed RA for 11 days using ThT fluorescence (ThT increases fluorescence intensity by binding to the β-sheet structure of aggregated Aβ^42^). In the group in which DA, DOPAC, NE, and L-DOPA coexisted with Aβ, the increase in fluorescence intensity was less compared with that in the group to which only Aβ was added. These results suggested that DA, DOPAC, NE, and L-DOPA had an inhibitory effect on Aβ aggregation at the concentrations used in this study. The concentration ratio of monoamines in the cerebral cortex, where aggregation inhibition was observed, was approximately 100:10:30:1 DA: DOPAC: NE: L-DOPA.

At present, the proposed mechanism of inhibition of Aβ aggregation by polyphenols and monoamines is via an *o*-quinone structure. Investigation of Aβ aggregation suppression by polyphenols such as Cur and RA has shown that the *o*-quinone structure specifically binds to Aβ, precluding further binding of Aβ to the oligomer^8^. The catechol group is oxidized to an *o*-quinone structure. Compounds with the *o*-quinone structure have been reported to covalently bind to nucleophilic amino acid residues or form Schiff bases and inhibit Aβ fibril extension^43^. DA is a representative compound in the catechol group, and its oxidation results in the *o*-quinone structure capable of bonding to the Aβ monomer for stabilization of protofibrils^44^. It has also been reported that the fiber elongation reaction is suppressed by destabilizing the terminal part of Aβ fibrils and accelerating depolymerization at the terminal^45^.

Raising DA concentration in the brain using RA may be useful in treating not only AD but also PD. PD is a neurodegenerative disease whose symptoms include body tremor and movement disorders. Although PD onset is unpredictable, the current theory suggests that a protein called α-synuclein accumulates abnormally in brain neurons^46^, and nerve cell death occurs in the SN, with the result that a decrease in the amount of DA in the striatum takes place^47^. Furthermore, since DA binds to α-synuclein and suppresses its accumulation^48^, elevation of intracerebral DA levels following RA feeding may be also effective against PD. Currently, selegiline taken orally is prescribed as an effective treatment for PD, acting to increase DA levels in the synaptic cleft. Another treatment method involves the administration of L-DOPA as a precursor of DA. L-DOPA can pass through the blood–brain barrier where it is metabolized to DA in the brain shortly after being administered^49^. However, both treatments have side effects including nausea and vomiting. In comparison, since RA is a familiar substance in the human diet, it should carry low risk as a treatment, and in fact, these negative effects were not observed in our animal study. Another type of dementia is dementia with Lewy bodies (DLB), thought to account for approximately 10% of dementia patients. Although the cause is unknown, inclusions called Lewy bodies are observed in neurons, predominantly in the brain stem and the cerebral cortex^50^. The major component of Lewy bodies is α-synuclein^51^, and abnormal accumulation of α-synuclein in the cerebral cortex is pathological. Thus, it is considered that raising intracerebral DA concentration by feeding RA could also assist treatment of DLB. Ho *et al*.^52^, reported that certain polyphenolic metabolites may attenuate α-synucleinopathy progression in the brain. Although we were unable to detect RA metabolites in this study, we plan to conduct a future study to clarify the relationship between RA metabolites and expression of monoamine-related enzymes genes.

In this study to elucidate anti-AD effects of RA intake, we found an increase in DA- related monoamine concentration in the cerebral cortex (Fig. 5b). In addition, we showed that the increase in the monoamine level in brain is likely caused by a decrease in the expression of *Maob* via inhibition of the expression of transcriptional factors, including *Tgf-β* and *Tieg2*. Further studies, such as those examining the time taken to elevate the concentration of monoamines and elucidating the regulation mechanism of *Maob* by RA, could build on these results to find application for clinical use.

## Methods

### Animals

For DNA microarray analysis, brain samples were obtained from the mice previously reported^9^. In brief, 5-month-old female Tg2576 mice (Taconic Farms, Germantown, NY) were used. They were divided into a control group fed a normal diet, and the RA group was fed an RA diet for 10 months. It was confirmed immunohistochemically for these mice that RA significantly decreased Aβ deposition in the brain. All animal studies were approved by the Institutional Animal Experiment Committee of Kanazawa University. For all other experiment, 7–10-week-old female C57BL/6 J mice (CLEA Japan Inc., Tokyo, Japan) were used. The mice were divided into two groups, a control (C) group fed with AIN-93G diet made in our laboratory from the necessary ingredients (Oriental Yeast Co., Shiga, Japan) as a normal diet, and an RA group with 0.5% RA added to the normal diet. All mice were housed individually in a room with a 12-h light/dark cycle with food and water *ad libitum*. All animal experiments were approved by the animal experiment committee of the University of Tokyo and performed in accordance with the relevant guidelines and regulations (approval number: P15-010 and P15–100).

### *In vivo* RA transferability test

The blood and whole brain were collected under isoflurane anesthesia after 7 weeks of breeding (C and RA group). The blood samples were centrifuged at 1,800 × *g* at 4 °C for 10 min to obtain EDTA plasma.

Phosphate-buffered saline (PBS) (1 mL/0.1 g of brain tissue) was added to the brain tissue sample and homogenized with a Polytron homogenizer. After that, the samples were centrifuged at 4,000 × *g* at 4 °C for 40 min to obtain the brain extract supernatant. The plasma and brain extract were processed as follows. In the β-glucuronidase-treated group, 50 μL of plasma or brain extract was suspended in 50 μL of 200 U β-glucuronidase and 0.1 M sodium acetate buffer (pH 5.0). Then the samples were incubated at 37 °C for 45 min. In the non-treated group, 50 μL of plasma or brain extract were suspended in 50 μL of 0.1 M sodium acetate buffer and incubated as above. After incubation, 200 μL of 0.83 M methyl acetate buffer was added to the samples of both groups, the protein was precipitated by vortex stirring for 1 minute, treated with sonication for 1 minute, and centrifuged at 8,500 × *g* at 4 °C for 5 min. The supernatants were recovered and passed through 0.45 μm filters, and the concentrations of RA and metabolites in the filtrates were measured using an Ultimate 3000 HPLC-ECD (Thermo Fisher scientific, Massachusetts, US). The conditions of chromatography were the same as the previous report^15^.

### BBB permeability of RA and metabolites

We measured the BBB permeability by using the BBB Kit (PharmaCo-Cell, Nagasaki, Japan) according to the manufacturer’s instructions. CA was used as a positive control, and Na-F as a negative control. The concentration of the assay buffer on the brain parenchyma side was measured by HPLC–ECD as per the *in vivo* RA transferability test. Na-F was measured with Ex/Em = 485/538 nm using a FlexStarion 3-fluorescence-plate reader (Molecular Devices, CA, USA). The permeability coefficient (Pe: × 10^−6^ cm/sec) was calculated as follows using the concentration of the test substance added to the luminal side ([C]_Luminal_), the concentration of test compound that passed through to the luminal side every 20 minutes ([C]_Abluminal_), and the amount on the brain parenchyma side (V_Abluminal_).

$${\rm{Volume}}\,(\mu {\rm{L}})=({[{\rm{C}}]}_{{\rm{Abluminal}}}\times {{\rm{V}}}_{{\rm{Abluminal}}})/{[{\rm{C}}]}_{{\rm{Luminal}}}$$

The amount of permeation was plotted every 20 min and the value of the slope of the formula of the quantity transmitted for each time was used as the permeation index (PS). In order to remove the influence of the membrane of the insert, the permeation index (PS_endothelial_) of the test substance was obtained from the following formula by subtracting the permeation index of the insert only (PS_membrane_) from the permeation index of the cell including the insert (PS_total_).

$${\rm{1}}/{{\rm{PS}}}_{{\rm{endothelial}}}={\rm{1}}/{{\rm{PS}}}_{{\rm{total}}}-{\rm{1}}/{{\rm{PS}}}_{{\rm{membrane}}}$$

P_e_ was calculated by dividing PS_endothelial_ by the insert surface area (A).

$${{\rm{P}}}_{{\rm{e}}}(\times {{\rm{10}}}^{-{\rm{6}}}{\rm{cm}}/\,\sec )={{\rm{PS}}}_{{\rm{endothelial}}}(\mu {\rm{L}}/{\rm{\min }})/{\rm{A}}\,{(\mathrm{cm}}^{{\rm{2}}})$$

### DNA microarray analysis in the brain of Tg2576 mice fed RA

The brain samples were obtained from the mice previously reported^9^. Total RNA was isolated from the frontal half of the left hemisphere using TRIzol (Thermo Fisher Scientific) according to the manufacturer’s instructions. The microarray analysis was conducted as described in Supplemental Methods.

### Monoamine concentration in the brain of mice fed RA for 7 weeks

The whole brain was collected under isoflurane anesthesia after 7 weeks of breeding (C and RA group). Buffer (2.0 mM NaHSO_3_, 20 μM EDTA • 2Na, 0.10 M HClO_4_; 1 mL/0.1 g of brain tissue) was added to the brain sample, the mixture was then homogenized with a Polytron and centrifuged at 12,000 × *g* at 4 °C for 5 min to obtain a brain extract supernatant. The supernatants were recovered, passed through 0.45 μm filters, and the concentration of monoamines in the filtrates was measured using HPLC-ECD. The conditions of chromatography were given in Supplemental Methods.

### DA imaging by IMS

Because it has been reported that isoflurane anesthesia increases the release of DA in rat striatum^53^, we adopted decapitation after cervical dislocation without anesthesia to euthanize the mice after 10 days of breeding (C and RA group). The left hemisphere of the brain of each mouse was collected, then sagittal sections 8 μm thick were prepared using a cryostat (CM-3050 S; LEICA, Germany) at −25 °C and mounted on a glass slide coated with indium tin oxide. Sections were sprayed with DPP and incubated at 40 °C for 30 min, and then sprayed with 2,5-dihydroxybenzoic acid to improve ionization efficiency. The surface of the sections was ionized by irradiation with laser light at 200 μm intervals using an Ultrafle Xtreme (matrix-assisted laser desorption/ionization time-of-flight mass spectrometer) (MALDI-TOFMS) (Bruker Daltonik GmbH, Bremen, Germany). The peak of DPP-derivatized DA (*m*/*z*: 368.2), which constitutes a complex of the target substance (DA) and DPP, was measured two-dimensionally. Only DA that could be derivatized was measured.

### Collection of SN and VTA from mouse brain

The mice were divided into two groups, C and RA, as mentioned above, and fed with the appropriate diet for 11 days. We collected SN and VTA from the brain according to methods previously described^54^. In brief, we collected the whole brain, washed it with saline, and obtained 1 mm thick coronal sections using a brain slicer BS-Z 2000C (Muromachi Kikai Co., Ltd., Tokyo, Japan). Referring to the Allen Brain Atlas^55^, we acquired the SN and VTA from the relevant sections.

### Relative mRNA measurement in cerebral cortex, SN, and VTA by qRT-PCR

Total RNA was extracted from SN, and VTA using TRIzol (Invitrogen) according to the manufacturer’s manual. After checking the quality of total RNA, we synthesized cDNA using PrimeScript^TM^ RT Master Mix (Perfect Real Time) (TaKaRa Bio, Shiga, Japan). The primer sequences and conditions for our qRT–PCR are given in Suppl. Table S1 and Supplemental Methods. Results are expressed as mean of fold change compared with control condition (SE as error bars, n = 5, 6). All samples were tested in triplicate and the data were normalized to GAPDH (cerebral cortex) or β-actin (SN and VTA).

### ThT assay

Dissolved lyophilized Aβ_1–42_ (PEPTIDE INSTITUTE, INC., Osaka, Japan) in 2 mM NaOH produced a peptide concentration of 0.5 mg/mL, which was then subjected to sonication by a bath-type sonicator for 1 min prior to re-lyophilizing the resulting solution. The NaOH-treated Aβ_1–42_ lyophilizate was dissolved in 500 μL distilled water and sonicated for 1 min. We added 500 μL of 20 mM phosphate buffer and centrifuged the resulting solution for 10 min at 16,000 × *g*. The supernatant was used as the Aβ_1–42_ solution. Aggregation of Aβ_1–42_ was measured using the SensoLyte® Thioflavin T β-Amyloid (1–42) Aggregation Kit (ANASPEC Inc., Fremont, CA.) according to the manufacturer’s protocol. To measure the Aβ_1–42_ fibril formation in the 96-well black microplates, 10 μL of 2 mM ThT and 5 μL of each monoamine solution (2 mM) were added into each well and mixed with 85 μL Aβ_1–42_ solution. The final concentration of Aβ_1–42_ was 45 μM and that of each monoamine concentration was 100 μM. The ThT fluorescence signal was monitored with Ex/Em = 440 nm/484 nm using Varioskan LUX (Thermo Fisher Scientific) at 37 °C at intervals of 5 min and 10 min for 0–125 min and 140–175 min, respectively.

All samples were tested in triplicate, and data were shown as relative fluorescence units by subtracting the fluorescence level of the blank from the actual value.

### Statistical analysis

Data were expressed as mean ± standard error. Statistical analyses were performed repeatedly using one-way or two-way analysis of variance, followed by student’s t and Tukey–Kramer or Dunnett’s tests, with a *P* value of < 0.05 considered as significant.

## Supplementary information

Dataset 1

## Data Availability

The datasets generated and/or analyzed during the current study are available from the corresponding author on reasonable request.
