# Supplementary material for: Rosmarinic acid suppresses Alzheimer’s disease development by reducing amyloid β aggregation by increasing monoamine secretion
Source: Sci Rep. 2019 Jun 18;9:8711. doi: 10.1038/s41598-019-45168-1 (PMC6581955; doi:10.1038/s41598-019-45168-1)
Supplement: Supplementary file 1 — Dataset 1 [file 41598_2019_45168_MOESM1_ESM.pdf]

Supplemental materials

Rosmarinic acid suppresses Alzheimer's disease development by reducing amyloid β aggregation by increasing monoamine secretion

Tomoki Hase[a], Syun Shishido[a], So Yamamoto[a], Rei Yamashita[a], Haruka Nukima[a], Shu Taira[b], Tsudoi Toyoda[c], Keiko Abe[c,d], Tsuyoshi Hamaguchi[e], Kenjiro Ono[f], Moeko Noguchi-Shinohara[e], Masahito Yamada[e], Shoko Kobayashi[a,]*

Methods

DNA microarray analysis

After that we purified total RNA using the RNeasy Mini Kit and RNase-Free DNase Set (Qiagen, Hilden, Germany). After synthesis of cDNA, biotin labeling, and fragmentation using GeneChip 3' IVT Express Kit (Thermo Fisher Scientific), each sample was hybridized onto GeneChip Mouse Genome 430 2.0 Array (Thermo Fisher scientific). The arrays were washed and labeled with streptavidin–phycoerythrin using a GeneChip Hybridization, Wash and Stain Kit and Fluidics Station 450 system (Thermo

Fisher scientific). Fluorescence was detected using a GeneChip Scanner 3000 7G (Thermo Fisher scientific). All experimental procedures were carried out according to the manufacturer's instructions. Affymetrix GeneChip Command Console software was used to convert the fluorescence values to the intensity of each probe (CEL files). The intensity values were analyzed using the statistics software program R (https://cran.r-project.org/) and Bioconductor (http://www.bioconductor.org/). We normalized the intensity values using the DFW method in R 2.7.2 and extracted probe sets that met FDR < 0.05 as DEGs by the Rank Products method using R 3.1.2. DEGs were annotated with GO terms using DAVID 6.8 (https://david.ncifcrf.gov), and GO terms with FDR of <0.05 were regarded as significantly enriched. FDR was calculated via the modified Fisher's exact test P value by the Benjamini and Hochberg method. The GO terms were listed hierarchically by QuickGO (https://www.ebi.ac.uk/QuickGO/). Ten KEGG pathways were selected in ascending order of modified Fisher's exact test P value.

qRT-PCR conditions

We performed qRT-PCR using SYBR® Premix Ex Taq™ (TaKaRa Bio) and Thermal Cycler Dice® Real-Time PCR System (TaKaRa Bio). Reaction conditions were as follows: 10 sec at 95°C for 1 cycle; 5 sec at 95°C + 30 sec at 60°C for 40 cycles; 15 sec at 95°C + 30 sec at 60°C + 15 sec at 95°C for 1 cycle.

The monoamine analytical conditions

We used an ODS column (150 mm length × 4.6 mm i.d.) (Thermo Fisher Scientific) with column temperature set at 40°C. Two mobile phases were used for gradient elution as follows. Buffer A: 0.10 M $Na_2HPO_4 \cdot 12H_2O$, 50 mM citric acid, 4.0 mM sodium 1-heptanesulfonate, 0.10 mM EDTA・2Na：MeCN：MeOH = 1000：25.9：62.9, and Buffer B: 0.10 M $Na_2HPO_4 \cdot 12H_2O$, 50 mM citric acid, 4.6 mM sodium 1-heptanesulfonate, 0.10 mM EDTA・2Na：MeCN：MeOH = 1000：77.7：188.7. Chromatography was carried out at a flow rate of 1.0 mL/min with a linear gradient under the following conditions: 0–14 min, Buffer A 100%; 14–23 min, Buffer B 0%–90%, 23–50 min, Buffer B 90%. Potential was detected with eight channels in total, from 0 to 560 mV with increments of 80 mV.

Supplementary Table S1. All primers sequences used in this study

Gene	Sequence (Forward)	Sequence (Reverse)
Th	5'-ACTGTCTCGGGCTTTGAAAGTG-3'	5'- ACCTCGAAGCGCACAAAGTAC-3'
Ddc	5'-ACCAGGACTCAGGATTCATCAC-3'	5'-ACGTGCTTTCGGATGTAAGC-3'
Comt	5'-CCCTTCGCACTGCCTGAG-3'	5'-ATGAGACAGCAGCCAACAGC-3'
Maob	5'-AATGGATGAGATGGGCCAAGAG-3'	5'-TCACAAAGAGCGTGGCAATC-3'
Dbh	5'-CTGACCGACTCAACTACTGC-3'	5'-CCGTGGGTTGTGGTAATGAAC-3'
Tieg2	5'-ACAAGAAGTTCGCAAGGTCAGATG-3'	5'- CGAGCGTGCTTTGTCAGGTGGTCA-3'
Tgf-β1	5′-TAATGGTGGACCGCAACAACG-3'	5′-TCCCGAATGTCTGACGTATTGAAG-3'
Gapdh	5'-GTCGGTGTGAACGGATTTGG-3'	5'-GACTCCACGACATACTCAGC-3'
β-actin	5'-GCTGACAGGATGCAGAAGGA-3'	5'-CTGGAAGGTGGACAGTGAGG-3'
